# Absolute quantification of DNA damage response proteins

**DOI:** 10.1186/s41021-023-00295-0

**Published:** 2023-12-18

**Authors:** Shun Matsuda, Tsuyoshi Ikura, Tomonari Matsuda

**Affiliations:** 1 Research Center for Environmental Quality Management, Kyoto University, 1-2, Yumihama, Otsu, Shiga 5200811 Japan; 2 Laboratory of Chromatin Regulatory Network, Department of Mutagenesis, Radiation Biology Center, Kyoto University, Yoshidakonoecho, Kyoto Sakyo-ku, Kyoto, 606-8501 Japan; 3 Laboratories for Environmental Health, Department of Environmental Engineering, Graduate School of Engineering, Kyoto University, Kyoto daigaku-katsura, Nishikyo-ku, Kyoto, 615-8530 Japan

**Keywords:** MDC1, γH2AX, Absolute quantification, LC-MS/MS, Chromatin affinity, DNA damage response

## Abstract

**Background:**

DNA damage response (DDR) and repair are vital for safeguarding genetic information and ensuring the survival and accurate transmission of genetic material. DNA damage, such as DNA double-strand breaks (DSBs), triggers a response where sensor proteins recognize DSBs. Information is transmitted to kinases, initiating a sequence resulting in the activation of the DNA damage response and recruitment of other DDR and repair proteins to the DSB site in a highly orderly sequence. Research has traditionally focused on individual protein functions and their order, with limited quantitative analysis, prompting this study’s attempt at absolute quantification of DNA damage response and repair proteins and capturing changes in protein chromatin affinity after DNA damage through biochemical fractionation methods.

**Results:**

To assess the intracellular levels of proteins involved in DDR and repair, multiple proteins associated with different functions were quantified in EPC2-hTERT cells. H2AX had the highest intracellular abundance (1.93 × 10^6^ molecules/cell). The components of the MRN complex were present at the comparable levels: 6.89 × 10^4^ (MRE11), 2.17 × 10^4^ (RAD50), and 2.35 × 10^4^ (NBS1) molecules/cell. MDC1 was present at 1.27 × 10^4^ molecules/cell. The intracellular levels of ATM and ATR kinases were relatively low: 555 and 4860 molecules/cell, respectively. The levels of cellular proteins involved in NHEJ (53BP1: 3.03 × 10^4^; XRCC5: 2.62 × 10^4^; XRCC6: 5.05 × 10^5^ molecules/cell) were more than an order of magnitude higher than that involved in HR (RAD51: 2500 molecules/cell). Furthermore, we analyzed the dynamics of MDC1 and γH2AX proteins in response to DNA damage induced by the unstable agent neocarzinostatin (NCS). Using cell biochemical fractionation, cells were collected and analyzed at different time points after NCS exposure. Results showed that γH2AX in chromatin fraction peaked at 1 h post-exposure and gradually decreased, while MDC1 translocated from the isotonic to the hypertonic fraction, peaking at 1 hour as well. The study suggests increased MDC1 affinity for chromatin through binding to γH2AX induced by DNA damage. The γH2AX-bound MDC1 (in the hypertonic fraction) to γH2AX ratio at 1 h post-exposure was 1:56.4, with lower MDC1 levels which may attributed to competition with other proteins.

**Conclusions:**

The approach provided quantitative insights into protein dynamics in DNA damage response.

## Introduction

DNA damage response (DDR) and DNA repair are crucial processes for organisms to protect genetic information and maintain the survival and accurate transmission of genetic material. Cells possess sensor proteins to detect DNA damage, recognizing abnormal DNA structures. Common sources of DNA damage include ultraviolet radiation, chemicals, and ionizing radiation, with one of the most severe forms being DNA double-strand breaks (DSBs). When DSBs occur in the cell, sensor proteins such as the MRN complex recognize them, transmitting this information to kinases like ATM and ATR, which phosphorylate the S139 of H2AX to form γH2AX and other proteins, resulting in activation of the DDR and recruitment of individual proteins to the DSB site in a highly orderly sequence [1]. The DNA damage response controls the cell cycle, halting it to repair damaged DNA and inducing cell death when necessary, preventing the erroneous replication of damaged DNA. DSBs are repaired through mechanisms such as non-homologous end joining (NHEJ) and homologous recombination (HR) [2], ensuring the accurate transmission of genetic information and the preservation of genetic integrity within organisms.

The study of these DDR and repair mechanisms has traditionally focused on the sequential functions of individual proteins, with limited quantitative (especially absolute quantitative) analysis. Therefore, in this study, we attempted the absolute quantification of DDR and repair proteins within cells. Additionally, by combining biochemical fractionation methods, we quantitatively captured changes in the chromatin affinity of proteins after DNA damage and report these findings.

## Materials and methods

### Cell culture

Human esophageal keratinocyte EPC2 cells immortalized by human telomerase reverse transcriptase [3] (EPC2-hTERT cells) (kindly provided by Dr. Hiroshi Nakagawa, University of Pennsylvania) were cultured in Keratinocyte-SFM (Life Technologies, Carlsbad, CA, USA) supplemented with 1 ng/mL epidermal growth factor (Life Technologies), 50 g/mL bovine pituitary extract (Life Technologies), 100 units/mL penicillin (Life Technologies) and 100 g/mL streptomycin (Life Technologies) at 37 °C in a humidified 5% CO2 incubator.

### DNA damage-induction by neocarzinostatin (NCS)

EPC2-hTERT cells (70% confluent in 100 mm dish) were treated with 200 ng/ml NCS (N9162, Sigma-Aldrich, St. Louis, MO, USA) for the indicated time. NCS solution was directly added to the medium. The cells were washed with phosphate-buffered saline (PBS) twice, collected, and suspended in 1 ml ice-cold PBS. Cell density was counted by a hemocytometer, cells were pelleted by centrifugation at 700×*g* for 5 min at 4 °C, and supernatant was removed. The cell pellet was instantaneously frozen with liquid nitrogen followed by thawing on ice.

### Cell fractionation by salt concentration

The cell pellet was resuspended in 500 μl hypotonic buffer (10 mM Tris-HCl pH 8.0, 1 mM KCl, 1.5 mM MgCl2, 1 mM dithiothreitol, 0.2 mM phenylmethylsulfonyl fluoride, and 10 mM β-glycerophosphate) and incubated on ice for 5 min. After centrifugation at 1300×*g* for 5 min at 4 °C, 150 μl of the supernatant was collected as the hypotonic fraction. After wash with 500 μl hypotonic buffer, the pellet was resuspended in 150 μl isotonic buffer (150 mM KCl, 1 mM dithiothreitol, 0.2 mM phenylmethylsulfonyl fluoride, and 10 mM β-glycerophosphate) and incubated on ice for 5 min. After centrifugation at 1300×*g* for 5 min at 4 °C, the supernatant was collected as the isotonic fraction. The pellet was resuspended in 150 μl hypertonic buffer (300 mM KCl, 1 mM dithiothreitol, 0.2 mM phenylmethylsulfonyl fluoride, and 10 mM β-glycerophosphate) and incubated on ice for 5 min. After centrifugation at 1700×*g* for 5 min at 4 °C, the supernatant was collected as the hypertonic fraction. The pellet was resuspended in 80 μl DNase I (Qiagen, Hilden, Germany) supplemented with 100 μg/ml RNase A (Qiagen) incubated at room temperature for 20 min. After addition of 80 μl 5 M NaCl solution (5 M NaCl, 1 mM dithiothreitol, 0.2 mM phenylmethylsulfonyl fluoride, and 10 mM β-glycerophosphate), the sample was rotated for 30 min at 4 °C and centrifuged at 16,000×*g* for 10 min at 4 °C. the supernatant and pellet were collected as the chromatin and insoluble fractions, respectively. Fifty microliters of 100% trichloroacetic acid was added to the hypotonic, isotonic, hypertonic, and chromatin fractions and the fractions were incubated on ice for 30 min. After centrifugation at 16,000×*g* for 10 min at 4 °C, the protein pellets were washed with 500 μl ice-cold acetone twice and air-dried. Two hundred microliters of 3% trichloroacetic acid was added to the insoluble fraction, and the fraction was incubated on ice for 30 min. After centrifugation at 16,000×*g* for 10 min at 4 °C, the protein pellets were washed with 500 μl ice-cold acetone twice and air-dried.

### Sodium dodecyl sulfate–polyacrylamide gel electrophoresis (SDS-PAGE)

The samples derived from non-treated cells were dissolved in 100 μl 8 M urea and equal volume of each sample was separated on NuPAGE 4–12% Bis-Tris Gel (Thermo Fisher Scientific, Waltham, MA, USA) and visualized by SimplyBlue SafeStain (Thermo Ficher Scientific), according to a manufacturer’s protocol.

### In-solution tryptic digestion

The samples were lysed in 105 μl 8 M urea supplemented with the mixture of internal standard peptides (Table 1) and diluted three times by 50 mM ammonium bicarbonate. After addition of 20 μl (the hypotonic and chromatin fractions) or 10 μl (the isotonic, hypertonic, and insoluble fraction) 0.1 μg/μl trypsin, the samples were incubated at 25 °C overnight. The samples were acidified by adding 10 μl 20% trifluoroacetic acid. Tryptic peptides were desalted on reversed phase C18 StageTips [4] and dissolved in 40 μl 80% dimethyl sulfoxide.

**Table 1:** Set of MRM transitions

Protein	Internal standard	Amino acid sequence of target peptide for quantification	MRM transition (*m*/*z* Q1 > Q3)	Collision energy (eV)
H2AX		ATQASQEY	449.2 > 526.2	10
γH2AX		ATQApSQEY	489.2 > 440.1	10
MDC1		VLFTGVVDAR	539.0 > 717.2	25
53BP1		VITDVYYVDGTEVER	344.6 > 459.5	11
NBS1		TTTPGPSLSQGVSVDEK	852.1 > 577.6	30
MRE11		GNDTFVTLDEILR	747.1 > 530.3	34
RAD50		GQDIEYIEIR	618.3 > 693.1	21
RAD51		YALLIVDSATALYR	785.3 > 461.5	24
ATR		APLNETGEVVNEK	700.3 > 875.5	23
ATM		YLNWDAVFR	592.3 > 492.6	22
RPA1		VIDQQNGLYR	603.5 > 622.5	22
XRCC5		EEASGSSVTAEEAK	698.0 > 834.5	22
XRCC6		SDSFENPVLQQHFR	852.4 > 512.9	32
H2AX	○	[^13^C_3_,^15^N]ATQASQEY	451.2 > 526.2	10
γH2AX	○	[^13^C_3_,^15^N]ATQApSQEY	491.2 > 442.1	10
MDC1	○	VLFTGVVDA[^13^C_6_,^15^N_4_]R	544.0 > 727.2	25
53BP1	○	VITDVYYVDGTEVE[^13^C_6_,^15^N_4_]R	349.5 > 469.5	11
NBS1	○	TTTPGPSLSQGVSVDE[^13^C_6_,^15^N_2_]K	856.1 > 585.6	30
MRE11	○	GNDTFVTLDEIL[^13^C_6_,^15^N_4_]R	752.1 > 540.3	34
RAD50	○	GQDIEYIEI[^13^C_6_,^15^N_4_]R	623.3 > 703.1	21
RAD51	○	YALLIVDSATALY[^13^C_6_,^15^N_4_]R	790.3 > 471.5	24
ATR	○	APLNETGEVVNE[^13^C_6_,^15^N_2_]K	704.3 > 883.5	23
ATM	○	YLNWDAVF[^13^C_6_,^15^N_4_]R	597.3 > 502.6	22
RPA1	○	VIDQQNGLY[^13^C_6_,^15^N_4_]R	608.5 > 632.5	22
XRCC5	○	EEASGSSVTAEEA[^13^C_6_,^15^N_2_]K	702.0 > 842.5	22
XRCC6	○	SDSFENPVLQQHF[^13^C_6_,^15^N_4_]R	857.4 > 522.9	32

### Liquid chromatography-tandem mass spectrometry (LC-MS/MS)

LC-MS/MS analysis followed a previous report with slight modification [5]. Mass spectrometric analysis was performed using a Xevo TQ-S (Waters, Manchester, UK) with an AQCUITY UPLC system (Waters). Five microliters of each sample was separated on an AQCUITY UPLC BEH C18 1.7 μm 2.1 × 50 mm column (Waters) at a flow rate of 0.5 ml/min, and subsequently eluted as follows (solvent A, 0.1% formic acid; solvent B, acetonitrile): for hydrophilic peptides, 0–0.5 min, isocratic with 10% B; 0.5–7 min, linear gradient to 30% B; 7–9 min, linear gradient to 80% B; 9–10 min, isocratic with 80% B; 10–12 min, isocratic with 10% B. for hydrophobic peptides, 0–0.5 min, isocratic with 20% B; 0.5–5 min, linear gradient to 30% B; 5–7 min, linear gradient to 80% B; 7–8 min, isocratic with 80% B; 8–10 min, isocratic with 20% B. MRM was performed in positive ion mode using nitrogen as the nebulizing gas. Experimental conditions were set as follows: ion source temperature, 150 °C; desolvation temperature, 650 °C; desolvation gas flow rate, 1000 L/h; capillary voltage, 2.51 kV; cone voltage, 30 V; cone gas flow rate, 150 L/h; collision gas, argon; collision gas flow rate, 0.15 ml/min. The conditions of multiple reaction monitoring (MRM) transitions, including collision energy, are shown in Table 1. The amount of each peptide was quantified by calculating the peak area ratio of the target peptide and its isotope-labeled internal standard. The calibration curve was obtained by using an authentic standard peptide spiked with its isotope-labeled internal standard.

## Results

### The intracellular levels of various DNA damage response and repair proteins

**Fig. 1:**
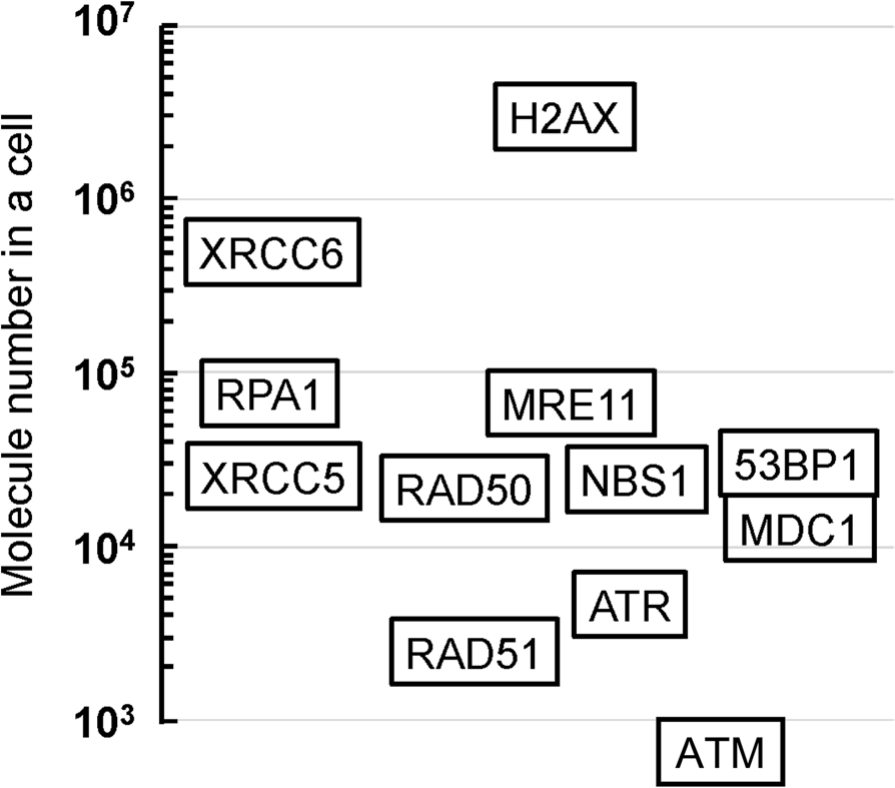
The abundance of each DNA damage response and repair protein in EPC2-hTERT cells

To assess the intracellular levels of proteins involved in DDR and repair, multiple proteins associated with different functions were quantified (Table 2 and Fig. 1). The variant of histone H2A, H2AX, had the highest intracellular abundance (1.93 × 10^6^ molecules/cell). The components of the MRN complex, which recognizes DSBs, were present at the following levels: 6.89 × 10^4^ molecules/cell (MRE11), 2.17 × 10^4^ molecules/cell (RAD50), and 2.35 × 10^4^ molecules/cell (NBS1). MDC1, acting as a platform binding to γH2AX and recruiting other DDR proteins to DSB sites [6], was present at 1.27 × 10^4^ molecules/cell. The intracellular levels of ATM and ATR kinases, which function in DDR, were 555 and 4860 molecules/cell, respectively. RPA1, recognizing single-stranded DNA, was present at 7.68 × 10^4^ molecules/cell, while proteins involved in NHEJ, 53BP1, XRCC5, and XRCC6, were present at 3.03 × 10^4^, 2.62 × 10^4^, and 5.05 × 10^5^ molecules/cell, respectively. The HR-related protein RAD51 was present at 2500 molecules/cell. Overall, the abundance of proteins involved in DNA damage response and repair varied significantly, spanning orders of magnitude greater than 10^3^.

**Table 2:** The abundance of each DNA damage response and repair protein in EPC2-hTERT cells

Protein	Molecule number in a cell (molecules/cell)	Protein	Molecule number in a cell (molecules/cell)
H2AX	1.93 × 10^6^	MDC1	1.27 × 10^4^
MRE11	6.89 × 10^4^	RPA1	7.68 × 10^4^
RAD50	2.17 × 10^4^	53BP1	3.03 × 10^4^
NBS1	2.35 × 10^4^	XRCC5	2.62 × 10^4^
ATM	555	XRCC6	5.05 × 10^5^
ATR	4860	RAD51	2500

### Cellular fractionation

**Fig. 2:**
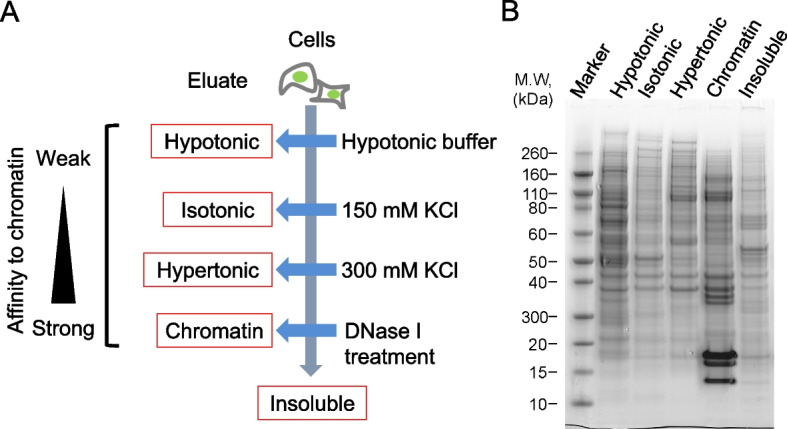
Cell fractionation. **a** Workflow of cell fractionation based on salt concentration in the buffer. **b** Coomassie staining of proteins in each fraction

Many DNA damage response and repair proteins are localized within the cell nucleus. Upon occurrence of DNA damage, these proteins accumulate at the damaged sites, leading to increased affinity for chromatin. The heightened chromatin affinity can be biochemically separated by exploiting the differential chromatin extraction capabilities based on salt concentration in the buffer [7]. To capture the dynamics of chromatin binding of proteins in response to DNA damage, cell fractionation was performed using variations in salt concentration in the buffer. The results of the fractionation, as visualized by SDS-PAGE, are presented in Fig. 2. The protein band profiles varied based on the salt concentration in the buffer, with prominent bands of histones observed in the chromatin fraction. Thus, it concluded that conditions for cellular fractionation had been established.

### The chromatin affinity dynamics of MDC1 following DNA damage

**Fig. 3:**
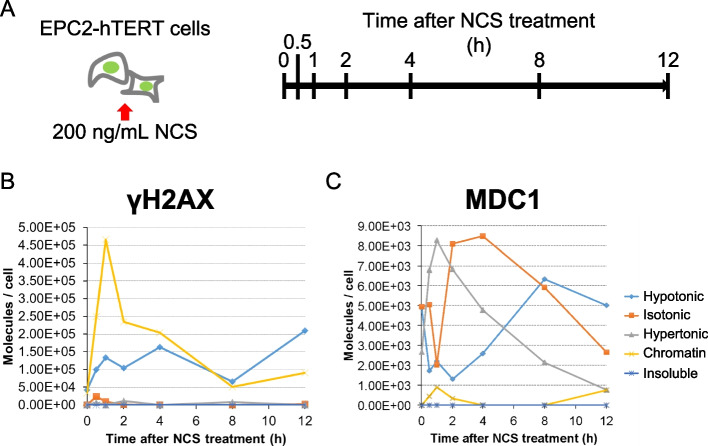
Dynamics of intracellular MDC1 chromatin affinity following DNA damage. **a** Exposure conditions of EPC2-hTERT cells to NCS. **b** Temporal changes in γH2AX levels in the chromatin fraction after NCS exposure. **c** Temporal changes in MDC1 levels in each fraction after NCS exposure

Focusing on MDC1 and γH2AX, we attempted to analyze the dynamics of these proteins following DNA damage using the aforementioned cell fractionation method. NCS is an unstable and potent DNA damage inducer, capable of inducing double-strand breaks in cells in a nearly pulse-like fashion within approximately 15 minutes of exposure [8]. Cells were collected and fractionated at specified time points after NCS exposure, and the temporal changes in the quantities of MDC1 in each fraction and γH2AX in the chromatin fraction were quantified. The results are presented in Fig. 3. γH2AX induced by NCS peaked at 1 hour after NCS exposure (4.66 × 10^5^ molecules/cell) and gradually decreased over the next 8 hours, reaching a plateau thereafter. MDC1, in the absence of exposure (0 h), was predominantly present in hypotonic (4870 molecules/cell) and isotonic fractions (5140 molecules/cell), with relatively fewer MDC1 molecules in the hypertonic fraction (2660 molecules/cell). MDC1 was not detected in the chromatin and insoluble fractions. Conversely, the amount of MDC1 in the hypertonic fraction peaked at 1 hour after NCS exposure (6790 molecules/cell), while MDC1 in the hypotonic and isotonic fractions decreased. Subsequently, the MDC1 level in the hypertonic fraction steadily decreased over 12 hours. MDC1 in the isotonic fraction increased from 2 to 4 hours and then steadily decreased over the next 12 hours, while MDC1 in the hypotonic fraction increased monotonically over 8 hours, reaching a plateau. Thus, we were able to quantitatively capture the dynamics of MDC1’s chromatin affinity in response to DNA damage.

## Discussion

In the context of cellular protein function, the control of expression levels is crucial. Interestingly, it has been observed that the intracellular molecular quantities of the components of the MRN complex, which functions as a DSB sensor—MRE11, RAD50, and NBS1—are approximately equivalent (2.17–6.89 × 10^4^ molecules/cell) (Fig. 1). MRN complex is known to be composed of two MRE11 subunits, two RAD50 subunits, and two NBS1 subunits [9]. MRE11, possessing nuclease activity [10], can pose a threat of dangerous DNA breaks to the cell when present alone. RAD50, by binding with MRE11, ATP-dependently inhibits the nuclease activity of MRE11 [11]. To facilitate a functional DNA damage response, it is reasonable to speculate that the expression levels of MRN complex components are cleverly regulated, ensuring the stability of the complex although the molecular abundance varies, to some extent, among these proteins within the cell. Furthermore, the intracellular expression levels of proteins involved in NHEJ (53BP1, XRCC5, and XRCC6) were more than an order of magnitude higher than those involved in HR (RAD51) (Fig. 1). While NHEJ occurs throughout the entire cell cycle, HR primarily takes place in the S phase [12]. Therefore, it seems reasonable that under conditions not synchronized with the cell cycle, as investigated in this study, many DSBs are repaired by NHEJ. Consequently, the higher expression levels of NHEJ proteins compared to HR proteins appear to be a rational outcome. In summary, the cellular expression levels of proteins have been suggested to be crucial factors in controlling DNA damage response and repair.

Exposure to NCS, which induces DSBs, resulted in an increase of γH2AX in the chromatin fraction, peaking at 1 h post-exposure (Fig. 3). Simultaneously, the translocation of MDC1 to the hypertonic fraction within the cell also peaked at 1 h post-NCS exposure. This suggests an increased affinity of MDC1 for chromatin through its binding to DSB-induced γH2AX. Considering the MDC1 in the hypertonic fraction binding to γH2AX (chromatin-bound type), the ratio of MDC1 to γH2AX at 1 h post-NCS exposure was 1:56.4. Taking into account the generation of γH2AX foci within a 2 Mbp DNA range by a single DSB [13], and considering that one nucleosome corresponds to 200 bp and that approximately 10% of histone H2A is H2AX, it is estimated that each focus generated by a single DSB contains approximately 1000 molecules of γH2AX and only about 20 molecules of MDC1. The lower number of MDC1 compared to γH2AX would be attributed to competition with other proteins possessing a BRCT domain that binds to γH2AX [14]. In summary, by combining biochemical fractionation with absolute quantification of proteins, the dynamics of chromatin affinity of proteins responding to DNA damage have been elucidated.

## Conclusions

In this study, we performed absolute quantification of DNA damage response and repair proteins and captured changes in protein chromatin affinity after DNA damage through biochemical fractionation methods, successfully. Our method and result provided quantitative insights into protein dynamics in DNA damage response, and be able to be applied to the other field.

## Data Availability

We declare that the data that support the findings of this study are available from the corresponding author upon reasonable request.

## Abbreviations

DDR: DNA damage response
DSB: DNA double-strand breaks
NCS: Neocarzinostatin
PBS: Phosphate-buffered saline
SDS-PAGE: Sodium dodecyl sulfate–polyacrylamide gel electrophoresis
LC-MS/MS: Liquid chromatography-tandem mass spectrometry
MRM: Multiple reaction monitoring
NHEJ: Non-homologous end joining
HR: Homologous recombination
